# Characterization of two constitutive promoters *RPS28* and* EIF1* for studying soybean growth, development, and symbiotic nodule development

**DOI:** 10.1007/s42994-022-00073-6

**Published:** 2022-06-13

**Authors:** Shengcai Chen, Yaqi Peng, Qi Lv, Jing Liu, Zhihua Wu, Haijiao Wang, Xuelu Wang

**Affiliations:** 1 College of Life Science and Technology, Huazhong Agricultural University, Wuhan, 430070 China; 2 State Key Laboratory of Crop Stress Adaptation and Improvement, School of Life Sciences, Key Laboratory of Plant Stress Biology, Henan University, Kaifeng, 475001 China; 3 Sanya Institute of Henan University, Sanya, 572025 China; 4 Hubei Provincial Key Laboratory for Protection and Application of Special Plant Germplasm in Wuling Area of China, Key Laboratory of State Ethnic Affairs Commission for Biological Technology, College of Life Sciences, South-Central University for Nationalities, Wuhan, 430074 China

**Keywords:** Promoter, Soybean (*Glycine max*), Ribosomal protein S28, Translation initiation factor EIF1, Constitutive expression

## Abstract

Native promoters that can drive high and stable transgene expression are important tools for modifying plant traits. Although several such promoters have been reported in soybean (*Glycine max*), few of them function at multiple growth and development stages and during nodule development. Here, we report that the promoters of *40S RIBOSOMAL PROTEIN SMALL SUBUNIT S28* (*RPS28*) and *EUKARYOTIC TRANSLATION INITIATION FACTOR 1* (*EIF1*) are ideal for high expression of transgene. Through bioinformatic analysis, we determined that *RPS28* and *EIF1* were highly expressed during soybean growth and development, nodule development, and various biotic and abiotic stresses. Fusion of both *RPS28* and *EIF1* promoters, with or without their first intron, with the reporter gene *β-GLUCURONIDASE* (*uidA*) in transgenic soybean, resulted in high GUS activity in seedlings, seeds, and nodules. Fluorimetric GUS assays showed that the *RPS28* promoter and the *EIF1* promoter yielded high expression, comparable to the soybean *Ubiquitin* (*GmUbi*) promoter. *RPS28* and *EIF1* promoters were also highly expressed in *Arabidopsis thaliana* and *Nicotiana benthamiana*. Our results indicate the potential of *RPS28* and *EIF1* promoters to facilitate future genetic engineering and breeding to improve the quality and yield of soybean, as well as in a wide variety of other plant species.

**Supplementary Information:**

The online version contains supplementary material available at 10.1007/s42994-022-00073-6.

## Introduction

Promoters are collections of *cis*-regulatory motifs and sequences located upstream of the transcription start site of genes that are recognized by transcription factors involved in transcription initiation (Dynan and Tjian 1985). The isolation and characterization of varied promoters suitable for plant genetic engineering based on their constitutive, tissue-specific, temporal, or inducible expression patterns is an essential step to introduce desirable traits into plants. In particular, promoters with high and constitutive expression are widely used in basic and applied research to control the expression of genes of interest in transgenic plants. However, the same promoter should not be used to drive the expression of separate transgenes in the same plant, as this may lead to transgene inactivation due to silencing (Que and Jorgensen 1998). Promoters from plant viruses, such as the cauliflower mosaic virus (CaMV) *35S* promoter and figwort mosaic virus (FMV) promoter, are routinely employed as constitutive promoters in plant research (Govindarajulu et al. 2008). However, they may drive the expression of transgene at levels in the wrong tissue or at the wrong time at a much higher level than the native plant promoters. Exploring efficient constitutive native promoters will be a critical step to develop transgenic plants that express the desired traits.

Soybean (*Glycine max* (L.) Merr.), which is a good source of protein and oil for human dietary needs, is a valuable agronomic crop worldwide. Soybean also can form a symbiotic relationship with *Rhizobium* species, producing nodules that can fix atmospheric nitrogen by converting it into ammonia for plant consumption in exchange for sugars (Yang et al. 2021). Despite its agronomic importance, the yield of soybean is relatively low, which has motivated extensive work on molecular breeding to overcome this limitation and produce new and improved transgenic soybean. Therefore, various strong native soybean promoters are required to drive transgene expression. Several promoters have been identified to direct expression in constitutive, tissue-specific, temporal, or inducible manners. For instance, 20 highly expressed soybean genes were identified using transcriptome deep sequencing (RNA-seq) data sets based on their high expression levels in one or more tissues or following a given treatment (Zhang et al. 2015). Their promoter activities were confirmed in lima bean (*Phaseolus lunatus*) cotyledons and soybean hairy roots. The activity of the promoters from two *ELONGATION FACTOR 1A* (e*EF1A*) genes was also detected in stable primary soybean transformants. Of the strong constitutive promoters employed to direct transgene expression in soybean, the *polyubiquitin* (*GmUbi*) promoter is the most extensively used (Grant et al. 2017). Several specific promoters have also been reported, such as the *HYPERSENSITIVE-INDUCED RESPONSE1* (*GmHIR1*) promoter expressed specifically in flowers and developing seeds (Koellhoffer et al. 2015), the pathogen-inducible *POLYPHENOL OXIDASE12* (*GmPPO12*) promoter (Chai et al. 2013), the heat- and 1-aminocyclopropane-1-carboxylic acid (ACC)-induced *TONOPLAST INTRINSIC PROTEIN2;6* (*GmTIP2;6*) promoter (Feng et al. 2019), the root-preferential *PROLINE-RICH PROTEIN2* (*GmPRP2*) promoter (Chen et al. 2014), and the abscisic acid (ABA)- and polyethylene glycol (PEG)-induced *RESPONSIVE TO DESICCATION26* (*GmRD26*) promoter (Freitas et al. 2019).

The promoters of reference genes typically used for normalization during quantitative PCR may also be used as constitutive promoters, as they are by definition evenly expressed across multiple tissues, developmental stages, or conditions. For instance, *CYCLOPHILIN2* (*GmCYP2*) and *UBIQUITIN-CONJUGATING ENZYME4* (*GmUBC4*) are stable reference genes expressed in different organs (roots, stems, leaves, flowers, and pods) (Miranda Vde et al. 2013). Similarly, *eEF1A* and *eEF1B* are the most stable reference genes across various developmental stages, including the third fully developed trifoliate leaf, full flowering, and developed pods. For spatial and temporal gene expression studies, *GmUBC4*, *GmUBC2*, *GmCYP2*, and *ACTIN11* (*GmACT11*) are considered as reference genes. *eEF1A* and *TUBULIN A5* (*GmTUA5*) are the most stable reference genes when studying gene expression of soybean roots infected with the roundworm *Meloidogyne incognita*. Likewise, *GmCYP2* and *eEF1A* are the most stable reference genes in soybean leaves under attack by velvetbean caterpillar (*Anticarsia gemmatalis*) (Miranda Vde et al. 2013). *ELONGATION FACTOR 1B* (*GmEF1B*) and *HYPOTHETICAL PROTEIN 2* (*UKN2*) are the best-suited reference genes in plants during virus infection (Ma et al. 2013), while *GmEF1A* and *GmACT11* are the best reference genes under high-salinity stress. Other genes have also been shown to exhibit stable expression under drought stress (*TUBULIN4* [*GmTUB4*], *GmTUA5*, and *GmEF1A*), in the dark (*GmACT11* and *GmUKN2*), or upon high salinity or drought stress and in the dark (*GmEF1B* and *GmUKN2*) (Ma et al. 2013). *GmEF1B* and *GmACT11* may be employed as references in O_2_-depleted environments, such as in flooded plants (Nakayama et al. 2014). However, the activities of these respective promoters have not been tested across the entire vegetative growth period, over multiple development stages, especially during nodule development. At the same time, the coefficient of variation of expression over the multiple tissues has not been evaluated.

In this study, we used bioinformatic analysis methods to search for native soybean promoters exhibiting strong and constitutive expression throughout vegetative growth and development as well as during nodule development. We selected ten candidate promoters and further tested two from the *40S RIBOSOMAL PROTEIN SMALL SUBUNIT S28* (*GmRPS28*, referred to as *RPS28* herein) and *EUKARYOTIC TRANSLATION INITIATION FACTOR 1* (*GmEIF1*, referred to as *EIF1* herein) genes. We generated transcriptional fusions between these two promoters and the *β-GLUCURONIDASE* (*uidA*) reporter gene and introduced them into soybean, Arabidopsis (*Arabidopsis thaliana*), and *Nicotiana benthamiana* for evaluation.

## Results

### Identifying highly expressed soybean genes from microarray and RNA-seq data

To identify genes with broad and high expression during plant growth and development, and nodule development, we first analyzed the expression levels of soybean genes from published 1072 microarray samples which generated with Affymetrix gene chips (Wu et al. 2019). We retained only those genes with an average value in the top 100 and coefficients of variation (CoV) less than 0.035, indicative of relatively constant expression across all samples (Table S1). To further remove the effect of environmental factors, we then ordered the retained 25 genes from published soybean RNA-sequencing samples (Machado et al. 2020; Table S2) on the basis of their minimum transcript per million (TPM) (mini TPM > 50) and CoV (CoV < 0.4) and kept the top 10 genes (Table 1). Among the top 10 genes, the ones which stably expressed under stress and have not been reported were selected as candidates. Gene 1 (Glyma.19G052400) and gene 6 (Glyma.15G050200) have been published previously (Yim et al. 2015; Bansal et al. 2015). Gene 2 (Glyma.19G172200) was upregulated by GmbZIPE2 overexpression. Gene 3 (Glyma.17G103100) and gene 7 (Glyma.05G024200) were reported to be induced by the soybean mosaic virus G7 strain (Chen et al. 2017). Gene 5 (Glyma.07G115900) encodes a nucleoside diphosphate kinase, which was reported to be regulated by NaCl-CaCl2 and NaCl-LaCl3 treatment (Yin et al. 2015). Gene 9 (Glyma.08G126200) was identified as a target gene of gma-miR5037c which is involved in salt stress and phosphate starvation (Ning et al. 2019; Tripathy et al. 2021). Gene 10 (Glyma.13G290900) was reported to be inhibited by drought stress (Wang et al. 2019). Finally, Glyma.19G203300 (hereafter as *RPS28*) and Glyma.06G269600 (hereafter *EIF1*) were selected for further characterization (Fig. S1A and B).

**Table 1:** Summary of the top 10 genes with high expression levels

	Gene ID	Min TPM	Max TPM	Mean TPM	CoV	Gene functional annotation
Gene 1	Glyma.19G052400	243.63	1153.50	649.67	0.37	Elongation factor Tu GTP binding domain
Gene 2	Glyma.19G172200	208.56	626.80	380.38	0.34	Hsp70 protein
Gene 3	Glyma.17G103100	176.96	718.89	442.64	0.28	Eukaryotic translation initiation factor 5A
Gene 4	Glyma.19G203300	164.94	582.13	293.99	0.35	40S ribosomal protein S28
Gene 5	Glyma.07G115900	145.20	562.65	340.46	0.32	Nucleoside diphosphate kinase
Gene 6	Glyma.15G050200	110.27	407.35	230.56	0.36	Actin
Gene 7	Glyma.05G024200	108.80	463.59	257.17	0.31	Eukaryotic translation initiation factor 5A
Gene 8	Glyma.06G269600	96.95	750.25	448.03	0.38	Eukaryotic translation initiation factor SUI1
Gene 9	Glyma.08G126200	66.30	175.41	115.36	0.29	RAB GDP-dissociation inhibitor
Gene 10	Glyma.13G290900	53.43	262.08	165.21	0.32	14-3-3 protein

*Min TPM* minimum transcript per million (TPM), *Max TPM* maximum TPM, *Mean TPM* average TPM, *CoV* Coefficient of Variation, SD/average TPM

### The *RPS28* and *EIF1* promoters contain multiple core and cis-acting elements

**Fig. 1:**
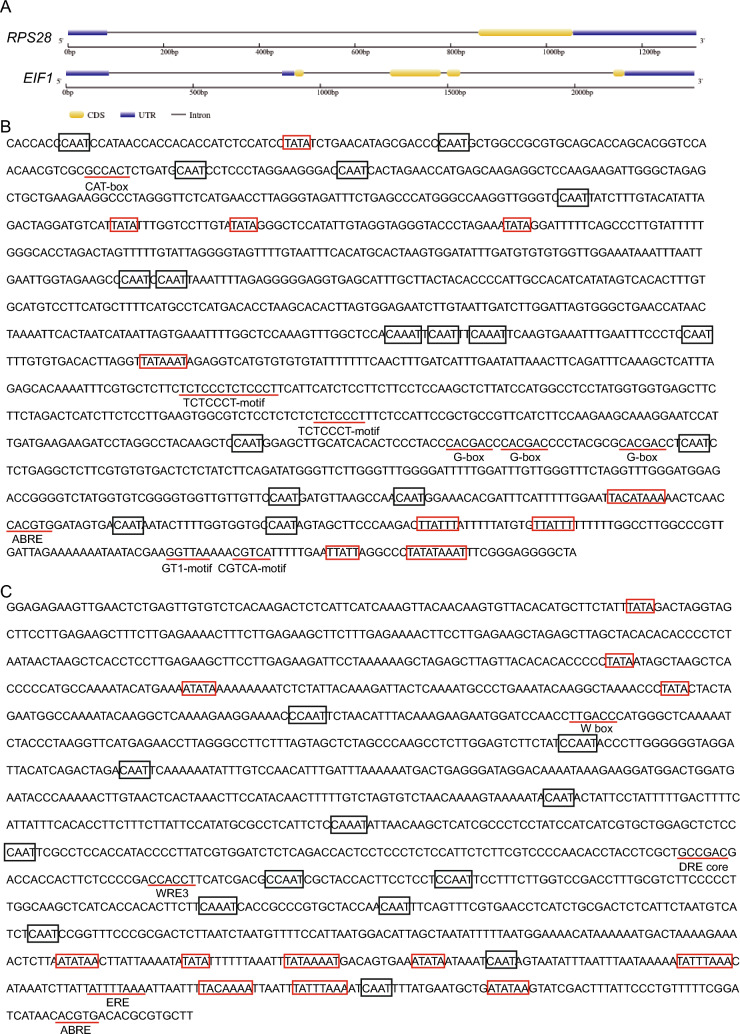
Promoter analysis of *RPS28* and *EIF1*. **A** Gene structures of *RPS28* and *EIF1*. **B** and **C** Promoter analysis of *RPS28* (**B**) and *EIF1* (**C**). The *cis*-acting elements are indicated as red (TATA-box) or black (CAAT-box) boxes or underlined in red (other *cis*-acting elements)

We next analyzed the gene structures of *RPS28* and *EIF1* (Fig. 1A). We found that the *RPS28* locus consists of a single exon separated from the 5' UTR by one large intron, while the *EIF1* locus comprises four introns, one of which was located in the 5' UTR. We then turned to the identification of the core promoter elements TATA-box and CAAT-box in the region upstream of the translational start site ATG using the databases PLACE (Higo et al. 1999) and PlantCARE (Lescot et al. 2002). We detected ten putative TATA-boxes and 17 putative CAAT-boxes in the *RPS28* promoter (Fig. 1B), and 12 putative TATA-boxes and 13 putative CAAT-boxes in the *EIF1* promoter (Fig. 1C). In addition, we observed additional putative *cis*-acting elements within the promoters, including the CGTCA motif, CAT-box, ABA-responsive element (ABRE), GT1 motif, G-box, and TCTCCCT motif in the *RPS28* promoter (Fig. 1B) and the ABRE, W-box, wound-responsive *cis*-element (WRE3), ethylene responsive element (ERE), and dehydration-responsive element (DRE) core in the *EIF1* promoter (Fig. 1C). These results suggested that the *RPS28* and *EIF1* promoters harbor both core and inducible promoter elements. To examine whether their expression levels were affected by these putative inducible cis-elements, we checked their expression levels under cold, drought, and salicylic acid (SA) treatment from a published soybean RNA-Seq database (Yu et al. 2022). The expression levels of these genes did not change significantly under different stress conditions (Fig. S2A–F; Table S3). Furthermore, *RPS28* and *EIF1* showed almost the same expression level compared with *GmUbi* (Fig. S2A–F). These results suggest that *RPS28* and *EIF1* are constitutively expressed genes under stress conditions, though there are some stress responsive cis-regulatory elements in their promoters.

### The *RPS28* and *EIF1* promoters drive high GUS gene expression at different growth and development stages in soybean

**Fig. 2:**
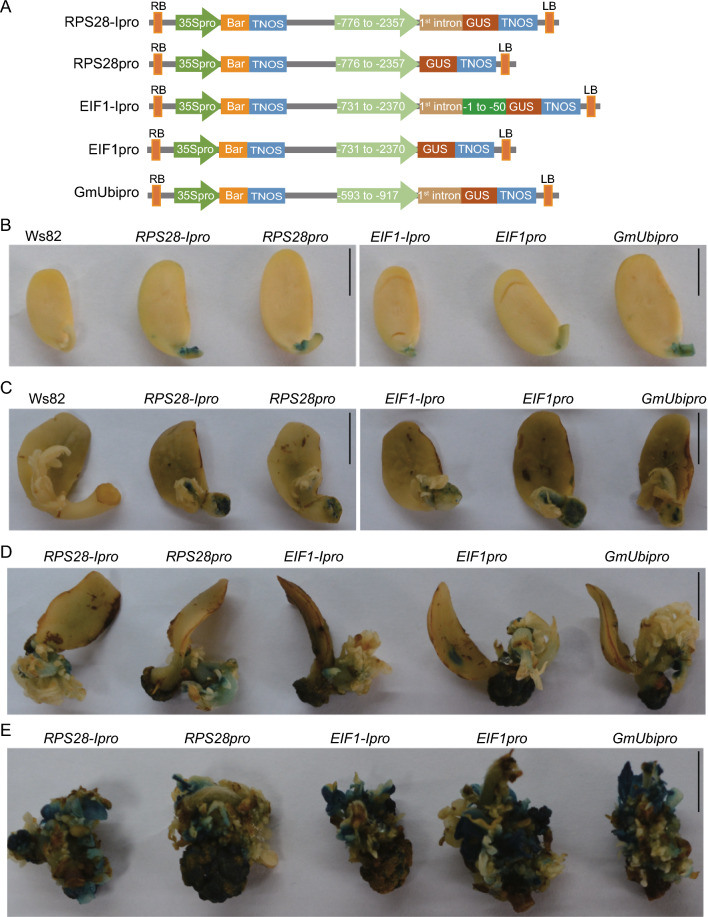
The *RPS28* and *EIF1* promoters drive *GUS* expression at different stages of soybean transformation. **A** Schematic representation of the binary vectors used for transformation. *RB* right border, *LB* left border, *Bar* phosphinothricin N-acetyltransferase, *TNOS,* nopaline synthase terminator. *RPS28pro*, *RPS28* promoter and 5′ UTR; *RPS28-Ipro*, *RPS28* promoter with 5′ UTR and the first intron; *EIF1pro* *EIF1* promoter and 5′ UTR; *EIF1-Ipro* *EIF1* promoter with 5′ UTR and first intron; *GmUbipro*, *Ubiquitin* promoter with 5′ UTR and the first intron (positive control). **B**–**E** *RPS28* and *EIF1* promoters support *GUS* expression during co-culture (**B**), 2 (**C**) and 4 weeks (**D**) after bud induction, and during bud elongation (**E**). In **B** and **C**, Ws82 cotyledon without infection was used as negative control. Scale bars = 1 cm

We next examined the tissue specificity of the *RPS28* and *EIF1* promoters in transgenic soybean plants. We first placed the *b-GLUCURONIDASE* (*GUS*) reporter gene under the control of the *RPS28* or *EIF1* promoters (1500 bp and 1472 bp, respectively) (Fig. 2A). For each promoter, we generated two reporter constructs: one consisting of the promoter and 5' UTR (*RPS28pro* and *EIF1pro*), and one also harboring the first intron (*RPS28-Ipro* and *EIF1-Ipro*), as studies have shown that the first intron also may influence gene expression (Le Hir et al. 2003). We also constructed another reporter containing the well-known constitutive promoter *GmUbi* with its intron in the 5' UTR as a positive control (Fig. 2A; Grant et al. 2017). We transformed Ws82 with the five constructs via Agrobacterium-mediated cotyledonary-node transformation and then analyzed their promoter activity in T_2_ stable transgenic lines.

**Fig. 3:**
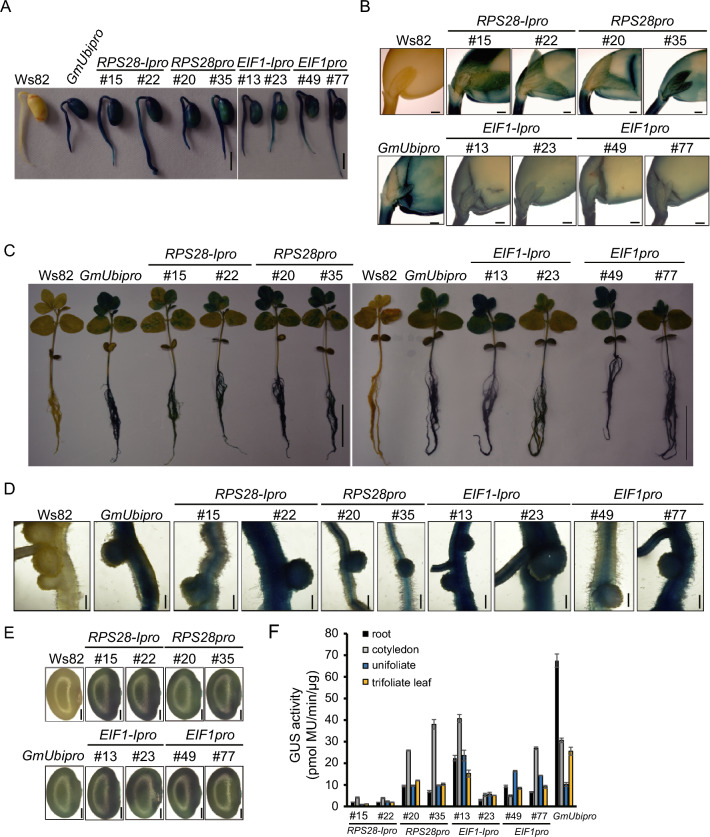
The *RPS28* and *EIF1* promoters drive *GUS* gene expression in transgenic soybean plants. **A** The 5-day-old seedlings. Scale bars = 1 cm. **B** Cotyledon with buds excised from the same 5-day-old seedlings shown in (**A**). Scale bars = 1 mm. **C** 15-day old seedlings. Scale bars = 10 cm. **D** Roots and nodules collected from 15 DPI. Scale bars = 0.5 mm. **E** Seeds at the filling stage. Scale bars = 1 mm. **F** Quantitative fluorimetric GUS assay in root, cotyledon, unifoliate, and trifoliate leaf of 15-day-old seedlings. Data are means ± standard deviation (SD)

We detected GUS enzyme activity by histochemical staining for all constructs during co-culture, bud induction, and bud elongation stages during transformation (Fig. 2B–E), indicating that these promoters may be useful to support transgene expression during stable transformation. To test if these constructs had any adverse effects on plant growth, we compared the growth phenotypes of stable transgenic lines with the wild-type Ws82 and found that all transgenic lines were comparable to the Ws82, indicating that GUS expression did not affect plant growth or development (Fig. S3A, B). In soybean transformants, we observed GUS activity derived from all constructs throughout the 5-day-old seedlings (Fig. 3A, B), and in the unifoliate leaf, trifoliate leaf, buds, petioles, internodes, roots, and nodules of 15-day-old plants (Fig. 3C, D, and Fig. S3C, D). We also detected GUS activity in reproductive tissues, such as young pods as well as pods and seeds at the seed-filling stage (Fig. 3E, and Fig. S3E, F). In addition, we quantified GUS activity with 4-methylumbelliferyl (4-MU) in the root, cotyledon, unifoliate leaf, and trifoliate leaf of 15-day-old transgenic soybean plants. Except *RPS28-Ipro*, *RPS28pro*, *EIF1-Ipro*, and *EIF1pro* showed almost similar GUS activity compared with *GmUbipro* in the root, cotyledon, unifoliate leaf, and trifoliate leaf (Fig. 3F). We concluded that the *RPS28* and *EIF1* promoters can confer constitutive highly and stably expression of transgenes in soybean and support transgene expression during transformation, growth and development, and in nodules with different expression levels.

### The *RPS28* and *EIF1* promoters drive GUS gene expressed in Arabidopsis and *Nicotiana benthamiana*

**Fig. 4:**
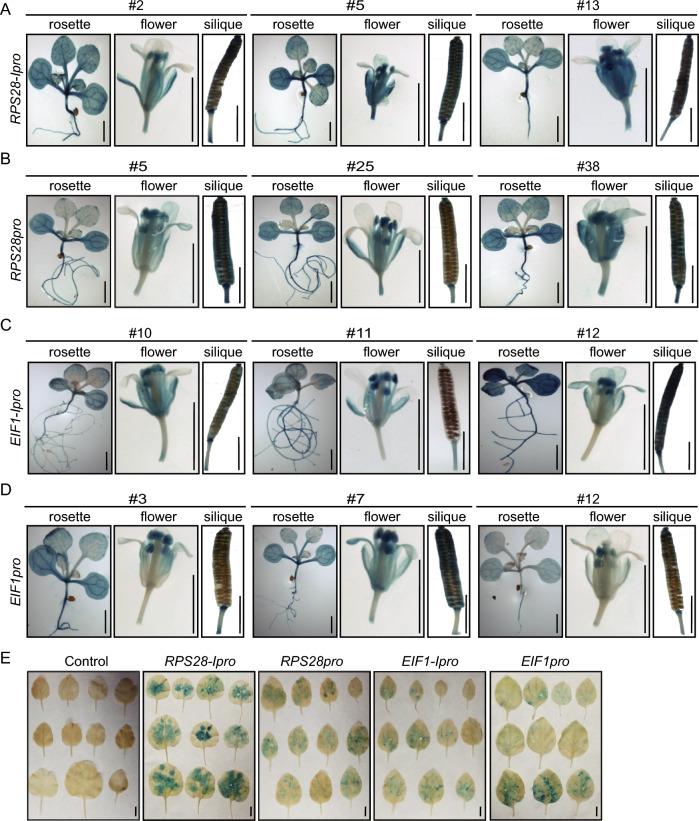
Representative GUS histochemical staining assay of transgenic Arabidopsis plants and *Nicotiana benthamiana* leaves driven by the *RPS28* and *EIF1* promoters. **A**, **B** *RPS28* promoter with the 5′ UTR and first intron (**A**) or 5′ UTR alone (**B**). **C**, **D** *EIF1* promoter with the 5′ UTR and first intron (**C**) or 5′ UTR alone (**D**). **E** GUS staining of the RPS28 and EIF1 promoters in *Nicotiana benthamiana* leaves. In **A**–**D**, GUS activity was tested in 7-day-old seedlings, flowers, and pods. In **A**–**D**, Scale bars = 2 mm. In **E**, Scale bars = 1 cm

We then tested whether the *RPS28* and *EIF1* promoters might also function in other species. To this end, we introduced the *RPS28pro:GUS*, *RPS28-Ipro:GUS*, *EIF1pro:GUS*, and *EIF1-Ipro:GUS* reporters into the model plants Arabidopsis and *N. benthamiana*. We observed GUS activity in almost all Arabidopsis tissues or organs, including seedlings, flowers, and pods (Fig. 4A–D). Likewise, we detected GUS activity in *N. benthamiana* leaves transiently transformed with the reporter constructs (Fig. 4E). Together, these results indicate that the *RPS28* and *EIF1* promoters can drive transgene expression in a variety of plant species.

## Discussion

Here, we reported two constitutive endogenous promoters derived from *RPS28* and *EIF1*. First, *RPS28* and *EIF1* were expressed in almost all soybean organs, as well as during nodule development, with a low coefficient of variation. Second, the *RPS28* and *EIF1* promoters exhibited high expression in stable soybean transformants. Third, the *RPS28* and *EIF1* promoters were both active in Arabidopsis and *N. benthamiana*.

*RPS28* and *EIF1* were not only constitutively expressed in different tissues and developmental stages and in nodules, but also under different conditions. Indeed, both genes were highly and stably expressed across 1072 different microarray datasets that covered 45 different genotypes and 18 tissues, as well as different biotic and abiotic stresses, such as rust disease, drought, aluminum stress, iron treatment, ABA treatment, and SA treatment (Wu et al. 2019). Although the presence of important *cis*-regulatory elements in the *RPS28* and *EIF1* promoters, they are highly expressed under different conditions. For instance, the *RPS28* promoter harbors ABRE element which is involved in ABA signaling (Yoshida et al. 2010); in the *EIF1* promoter, besides ABRE, we identified W-box motifs, which are bound by WRKY transcription factors and are related to drought stress (Li et al. 2020), and DRE elements, which activate transcription in response to both low temperature and water deficit (Knight et al. 1999). We found that RPS28 and EIF1 constitutively expressed under cold, drought, and water deficit conditions.

The inclusion of introns in transgenes can dramatically affect the expression outcome (Le Hir et al. 2003). Although a *GmUbi* promoter construct without an intron still drives transgene expression to levels over two times greater than the CaMV *35S* promoter, the intron in the 5' UTR of the *GmUbi* promoter contributes to the highest activity of this promoter (Chiera et al. 2007; Grant et al. 2017). Similarly, the first intron of soybean *eEF1A* also is essential for the high activity of this native promoter (Zhang et al. 2016). Based on our analysis of the *RPS28* and *EIF1* loci, the 5′ UTR of *RPS28* is followed by a large intron, while the *EIF1* 5′ UTR is interrupted by a large intron. Notably, the omission of the intron from the *RPS28* promoter construct resulted in enhanced expression in root, cotyledon, unifoliate leaf, and trifoliate leaf. *RPS28* was independently screened as being highly expressed across multiple tissues, but it showed a low expression level in the latter evaluation (Zhang et al. 2015). In the paper of Zhang et al., the first intron of *RPS28* was included in the promoter sequence, which is similar to *RPS28-Ipro* in our study. The promoter activity of *RPS28* was evaluated in lima bean cotyledons and soybean hairy root (Zhang et al. 2015). In our study, the expression level of *RPS28-Ipro* is the lowest in cotyledon and root, which is consistent with Zhang et al. The first intron may be the reason for the low activity of *RPS28* promoter in their paper. However, the presence of the *RPS28* leader intron is pivotal under some conditions, as we observed much stronger activity from the *RPS28-Ipro* reporter construct in *N. benthamiana* leaves relative to the *RPS28pro* construct.

In summary, we described the two highly expressed constitutive *RPS28* and *EIF1* promoters in soybean. Both promoters drove strong and constant expression during plant growth and development and nodule development, and under several biotic and abiotic stress conditions, and should serve as efficient promoters to generate transgenic plants with stable quality and yield. The two promoters have at least two advantages compared with *GmUbi*. First, the expressions of *RPS28* and *EIF1* are more stable than *GmUbi* indicated by the Coefficients of Variation (CoV) from 1072 microarray samples (table S1). Second, different promoters are necessary to drive the expression of multiple genes of interest in the same plant, as using a single promoter multiple times in one plant may lead to transgene silencing. Thus, the *RPS28* and *EIF1* promoters can be used as candidate promoters when several genes were constructed in one construct.

## Materials and methods

### Plant materials and growth conditions

The soybean ecotype ‘Williams 82’ and transgenic plants were grown in a greenhouse at 28 °C, under a 12 h light/12 h dark photoperiod. For nodulation assays, seeds were surface-sterilized with chlorine gas for 16–20 h and sown in wet vermiculite. After ~ 8 days, seedlings were inoculated with 2 mL of the *Rhizobium* strain *Bradyrhizobium diazoefficiens* USDA110 at an OD_600_ of ~ 0.1. Arabidopsis seedlings were grown on half-strength Murashige and Skoog (MS) medium for 7–8 days and then transferred to soil at 23 °C under a 16 h light/8 h dark photoperiod. *Nicotiana benthamiana* plants were grown at 26 °C under a 16 h light/8 h dark photoperiod.

### Gene structure and promoter analysis

The gene structures of *RPS28* and *EIF1* were deduced using the Gene Structure Display Server (GSDS2.0, http://gsds.cbi.pku.edu.cn/index.php). *cis*-Acting elements were predicted using the PLACE (http://www.dna.affrc.go.jp/PLACE/) and PlantCARE (http://bioinformatics.psb.ugent.be/webtools/plantcare/html/) online databases.

### Vector construction

Upstream regulatory sequences of the *PRS28* and *EIF1* genes are downloaded from Phytozome (Goodstein et al. 2012). The respective promoter fragments from genomic DNA of the soybean ecotype ‘Ws82’ were then PCR-amplified with specific primers (Table S4). Specifically, the PCR amplicon for the *RPS28* promoter (*RPS28pro*) included 1500 bp of promoter and 82 bp of 5′ untranslated region (5′ UTR); a promoter fragment with the first intron of RPS28, for a total amplicon size of 2357 bp (*RPS28-Ipro*), was also generated. The *EIF1* promoter was amplified as a 1640-bp amplicon comprising 1472 bp of promoter and 168 bp of 5′ UTR (*EIF1pro*) or with an additional 730 bp corresponding to the first intron (*EIF1-Ipro*). All PCR amplicons were cloned upstream of *uidA* in the vector pCAMBIA-1391Z harboring the *phosphinothricin acetyltransferase* (*bar*) gene as selection marker. Similarly, the *GmUbi* promoter (Glyma.20G141600) was cloned as a 917-bp amplicon covering the promoter, 5′ UTR, and first intron upstream of *uidA* in pCAMBIA-1391Z vector as positive control. For transformation in Arabidopsis and *Nicotiana benthamiana*, the original selection marker in pCAMBIA-1391Z, *Hygromycin B phosphotransferase* (*HygR*), was used.

### Soybean stable transformation

Stable transformation of soybean was performed following the previously reported cotyledonary-node transformation method (Luth et al. 2015).

### Arabidopsis transformation assay and transient expression assays in Nicotiana benthamiana leaves

The corresponding constructs were introduced into Agrobacterium (*Agrobacterium tumefaciens*) strain GV3101 and cultured on solid medium containing 100 μg/mL kanamycin. Single colonies were grown in liquid medium with 100 μg/mL kanamycin at 28 °C for 2 days. After centrifugation, the cell pellets were resuspended to a final OD_600_ ≈1.0 in transformation buffer (10 mM MES pH 5.7, 10 mM MgCl_2_, and 200 μM acetosyringone). For Arabidopsis transformation, the prepared strain liquid was used to transform plants by the floral-dip method (Clough and Bent 1998). Transgenic plants were screened on 0.5xMS plates supplemented with 35 mg/L Hygromycin. For *N. benthamiana* transient expression assay, the prepared strain liquid was infiltrated into young *N. benthamiana* leaves. After an incubation for 24 h in the dark, *N. benthamiana* plants were allowed to grow for another 36–48 h under a 16 h light/8 h dark photoperiod, and then, the leaves were collected and stained for GUS activity.

### Histochemical and fluorimetric GUS assay

For histochemical GUS assays, samples were soaked in 90% (v/v) acetone for 30 min for fixation and incubated in 1 mg/mL 5-bromo-4-chloro-3-indolyl-β-D-glucuronic acid, cyclohexylammonium salt (X-Gluc) solution overnight at 37 °C in the dark. After removal of the X-Gluc solution, samples were destained in 70% (v/v) ethanol. Photographs were taken using a digital camera or stereo light microscope (Zeiss V20). For fluorimetric GUS assays, GUS activity in root, cotyledon, unifoliate, and trifoliate leaf of 15-day-old seedlings was measured as previously described (Freitas et al. 2019).

### Supplementary Information

Below is the link to the electronic supplementary material.

Supplementary file1 (DOCX 2154 KB)

Supplementary file2 (XLSX 1320 KB)

Supplementary file3 (XLSX 47 KB)

Supplementary file4 (XLSX 22 KB)

## Data Availability

The datasets generated during and/or analysed during the current study are available from the corresponding author on reasonable request.
