# Correction: Characterization of two constitutive promoters *RPS28* and* EIF1* for studying soybean growth, development, and symbiotic nodule development

**DOI:** 10.1007/s42994-024-00171-7

**Published:** 2024-06-24

**Authors:** Shengcai Chen, Yaqi Peng, Qi Lv, Jing Liu, Zhihua Wu, Haijiao Wang, Xuelu Wang

**Affiliations:** 1 College of Life Science and Technology, Huazhong Agricultural University, Wuhan, 430070 China; 2 State Key Laboratory of Crop Stress Adaptation and Improvement, School of Life Sciences, Key Laboratory of Plant Stress Biology, Henan University, Kaifeng, 475001 China; 3 Sanya Institute of Henan University, Sanya, 572025 China; 4 Hubei Provincial Key Laboratory for Protection and Application of Special Plant Germplasm in Wuling Area of China, Key Laboratory of State Ethnic Affairs Commission for Biological Technology, College of Life Sciences, South-Central University for Nationalities, Wuhan, 430074 China

**Correction: aBIOTECH (2022) 3:99-109** 10.1007/s42994-022-00073-6

The article “Characterization of two constitutive promoters RPS28 and EIF1 for studying soybean growth, development, and symbiotic nodule development”, written by Shengcai Chen, Yaqi Peng, Qi Lv, Jing Liu, Zhihua Wu, Haijiao Wang, Xuelu Wang, was originally published Online First without Open Access. After publication in volume 3, issue 2, page 99–109 the author decided to opt for Open Choice and to make the article an Open Access publication. Therefore, the copyright of the article has been changed to © The Authors 2024 and the article is forthwith distributed under the terms of the Creative Commons Attribution CC BY 4.0 International License, which permits use, sharing, adaptation, distribution and reproduction in any medium or format, as long as you give appropriate credit to the original author(s) and the source, provide a link to the Creative Commons licence, and indicate if changes were made.

The images or other third party material in this article are included in the article’s Creative Commons licence, unless indicated otherwise in a credit line to the material. If material is not included in the article’s Creative Commons licence and your intended use is not permitted by statutory regulation or exceeds the permitted use, you will need to obtain permission directly from the copyright holder.

To view a copy of this licence, visit http://creativecommons.org/licenses/by/4.0/.

