# Supplementary material for: Characterization of two constitutive promoters RPS28 and EIF1 for studying soybean growth, development, and symbiotic nodule development
Source: aBIOTECH. 2022 Jun 13;3(2):99–109. doi: 10.1007/s42994-022-00073-6 (PMC9590564; doi:10.1007/s42994-022-00073-6)
Supplement: Supplementary file 1 — Supplementary file1 (DOCX 2154 KB) [file 42994_2022_73_MOESM1_ESM.docx]

**Supplementary material for**

**Characterization of two constitutive promoters *RPS28* and *EIF1* for studying soybean growth, development and symbiotic nodule development**

Shengcai Chen^1,5^, Yaqi Peng^2,3,5^, Qi Lv^2,3^, Jing Liu^1,2,3^, Zhihua Wu^4^, Haijiao Wang^2,3,*^ and Xuelu Wang^2,3,*^

^1^College of Life Science and Technology, Huazhong Agricultural University, Wuhan 430070, China

^2^State Key Laboratory of Crop Stress Adaptation and Improvement, School of Life Sciences, Key Laboratory of Plant Stress Biology, Henan University, Kaifeng 475001, China

^3^Sanya Institute of Henan University, Sanya, Hainan, 572025, China

^4^Hubei Provincial Key Laboratory for Protection and Application of Special Plant Germplasm in Wuling Area of China, Key Laboratory of State Ethnic Affairs Commission for Biological Technology, College of Life Sciences, South-Central University for Nationalities, Wuhan, China

^5^These authors contributed equally.

^*^Correspondence: Xuelu Wang (xueluw@henu.edu.cn); Haijiao Wang (wanghaijiao@henu.edu.cn)

**Fig. S1.** **The *RPS28* and *EIF1* genes constitutively expressed in soybean.**

(**A**) and (**B**) Relative expression levels of *RPS28* (**A**) and *EIF1* (**B**) in multiple tissues across RNA-seq samples (data download from https://venanciogroup.uenf.br/cgi-bin/gmax_atlas/).

**Fig. S2. RPS28 and EIF1 constitutively expressed in different stress conditions.** (A-F) the expression level of RPS28, EIF1, and GmUbi in unifoliate leaf after cold treatment (A), leaf and root under 8% PEG8000 treatment (B), leaf after water deficit (C-D), leaf and root under NaCl treatment (E), and shoot apex after SA treatment (F). In (A-C) and (E-F), the experiment was carried out under Williams 82 ecotype. In (D), Magellan was used as the ecotype. The data was download from http://ipf.sustech.edu.cn/pub/soybean/.

**Fig. S3. Histochemical localization of GUS activity in transgenic soybean plants expressing GUS driven by the *RPS28* and *EIF1* promoters.**

(**A**, **B**) Phenotypes of 15-day-old plants expressing *GUS* under the control of *RPS28-Ipro* and *RPS28pro* (**A**) or *EIF1-Ipro* and *EIF1pro* (**B**).

(**C**, **D**) GUS staining of unifoliate, trifoliate leaf, buds, petioles, and internodes of 15-day-old plants expressing *GUS* under the control of the *RPS28* (**C**) or *EIF1* promoter (**D**). Scale bars = 1 cm.

(**E**, **F**) GUS staining of young pods (top) and pods at the seed-filling stage (bottom) in plants expressing *GUS* under the control of the *RPS28* (**E**) or *EIF1* promoter (**F**). Scale bars = 1 cm.

**Table S4. Primer sequences used in this study.**

| Primer Name | Primer sequence (5’-3’) | note |
| --- | --- | --- |
| RPS28pro-F | GACCATGATTACGCCAAGCTT  CACCACCCAATCCATAACCACCAC | Cloning RPS28 promoter with the 5’-UTR into vectors |
| RPS28pro-R | CCAGTGAATTCCCGGGGATCC  CTGATGCAAAACACGAACAAAGAAAG |  |
| RPS28-Ipro-R | CCAGTGAATTCCCGGGGATCC  CCTGCTCAAACACAATCAACAG | Cloning RPS28 promoter with the 5’-UTR and first intron into vectors; the forward primer is RPS28pro-F |
| EIF1pro-F | GACCATGATTACGCCAAGCTT  GGAGAGAAGTTGAACTCTGAGTTGTG | Cloning eIF1 promoter with the 5’-UTR into vectors |
| EIF1pro-R | CCAGTGAATTCCCGGGGATCC  CTGATCGTAAATTTAAGGTTTCG |  |
| EIF1-Ipro-R | CCAGTGAATTCCCGGGGATCC  AAAACTTGACTCACTAAGACCAAAGG | Cloning eIF1 promoter with the 5’-UTR and first intron into vectors, the forward primer is EIF1pro-F |
| GmUbipro-F | ttacgccaagcttggctgcag  GGGCCCAATATAACAACGAC | Cloning GmUbiquitin promoter with the 5’-UTR and first intron into vectors |
| GmUbipro-R | CCAGTGAATTCCCGGGGATCC  ctgtcgagtcaacaatcaca |  |
| Bar-F | ATGAGCCCAGAACGACGCCCGGCC | Verification of positive transgenic soybean lines |
| Bar-R | TTAGATCTCGGTGACGGGCAGGAC |  |
